# Non-invasive mapping of human placenta microenvironments throughout pregnancy with diffusion-relaxation MRI

**DOI:** 10.1016/j.placenta.2023.11.002

**Published:** 2023-11-07

**Authors:** Paddy J. Slator, Daniel Cromb, Laurence H. Jackson, Alison Ho, Serena J. Counsell, Lisa Story, Lucy C. Chappell, Mary Rutherford, Joseph V. Hajnal, Jana Hutter, Daniel C. Alexander

**Affiliations:** a Cardiff University Brain Research Imaging Centre, School of Psychology, Maindy Road, Cardiff, CF24 4HQ, UK; b School of Computer Science and Informatics, Cardiff University, Cardiff, UK; c Centre for Medical Image Computing and Department of Computer Science, University College London, London, UK; d Centre for the Developing Brain, School of Biomedical Engineering and Imaging Sciences, King’s College London, London, UK; e Biomedical Engineering Department, School of Biomedical Engineering and Imaging Sciences, King’s College London, London, UK; f Department of Women and Children’s Health, School of Life Course Sciences, King’s College London, London, UK

**Keywords:** Placenta, Diffusion-relaxation MRI, Unsupervised learning, Diffusion MRI

## Abstract

**Introduction:**

In-vivo measurements of placental structure and function have the potential to improve prediction, diagnosis, and treatment planning for a wide range of pregnancy complications, such as fetal growth restriction and pre-eclampsia, and hence inform clinical decision making, ultimately improving patient outcomes. MRI is emerging as a technique with increased sensitivity to placental structure and function compared to the current clinical standard, ultrasound.

**Methods:**

We demonstrate and evaluate a combined diffusion-relaxation MRI acquisition and analysis pipeline on a sizable cohort of 78 normal pregnancies with gestational ages ranging from 15 + 5 to 38 + 4 weeks. Our acquisition comprises a combined T2*-diffusion MRI acquisition sequence - which is simultaneously sensitive to oxygenation, microstructure and microcirculation. We analyse our scans with a data-driven unsupervised machine learning technique, InSpect, that parsimoniously identifies distinct components in the data.

**Results:**

We identify and map seven potential placental microenvironments and reveal detailed insights into multiple microstructural and microcirculatory features of the placenta, and assess their trends across gestation.

**Discussion:**

By demonstrating direct observation of micro-scale placental structure and function, and revealing clear trends across pregnancy, our work contributes towards the development of robust imaging biomarkers for pregnancy complications and the ultimate goal of a normative model of placental development.

## 1 Introduction

Many common pregnancy complications, such as fetal growth restriction (FGR), pre-eclampsia, and stillbirth, are primarily associated with placental dysfunction [8]. Non-invasive biomarkers of small-scale human placental structure and function during pregnancy can lead to increased understanding, and hence potentially identify the origins of placental dysfunction. This can ultimately contribute to new techniques suitable for the prediction, diagnosis, and monitoring of pregnancy complications.

Multiple placental MRI methods are emerging as promising non-invasive imaging techniques for pregnancy assessment. One such method is quantitative T2* mapping, which calculates a T2* value in each image voxel by fitting a model to multiple gradient echo images with varying echo time (TE). This indirectly measures tissue oxygenation along with tissue density and magnetic field inhomogeneities. T2* mapping effectively detects tissue changes during pregnancy, as previous studies indicate T2* decreases over gestation [20]. T2* has also been found to be lower in cases of fetal growth restriction (FGR) [16,21,37] and preeclampsia [18,20], showing its potential for evaluating pregnancy complications.

Another sensitive imaging technique is diffusion MRI (dMRI), which measures the diffusion of water molecules in tissues. This allows for the quantification of sub-voxel microstructure by using a specialised diffusion-weighted MRI sequence and mathematical modelling. Consequently, this method can measure parameters related to tissue micro-structure and microcirculation with sensitivity to structures much smaller than the voxel size. dMRI has been applied to the placenta, demonstrating its sensitivity to microstructural changes over gestation. The apparent diffusion coefficient (ADC), which estimates how fast water is diffusing within tissue, decreases over gestation [17,20]. The IVIM perfusion fraction, which estimates the volume of perfusing blood, shows varying results, with some studies indicating an increase [33,36, 43,50], and others showing a decrease [11,23,26] over gestation. Moreover, dMRI is sensitive to placental dysfunction, with lower ADC values observed in cases of FGR [7,15,46]. The IVIM perfusion fraction is also altered in pregnancy complications [2,14,25,28–30,33,44]. These findings highlight the potential of dMRI as a valuable tool for assessing various aspects of placental health during pregnancy.

A limitation of these T2* and ADC approaches is that T2* and diffusion properties interact and influence each other, but are currently measured separately in the majority of cases [42]. This motivates a promising trend in microstructure imaging, combined diffusion-relaxation MRI, which has been applied multiple times in the placenta. Here the relevant MRI acquisition parameters for diffusion (e.g. b-values and gradient directions) and relaxation (e.g. TEs) are simultaneously varied to yield sensitivity to diffusion and relaxation properties and the correlations between them. Examples include combined T2-diffusion, achieved by acquiring dMRI data with multiple spin echoes with varying TE, e.g. Ref. [32], and combined T2*-diffusion imaging, by appending gradient echoes to a diffusion-encoding experiment [19]. This data, when combined with appropriate modelling approaches, can yield more sensitive measurements of tissue microstructure. Combined diffusion-relaxation placental MRI is attractive as it has the potential to disentangle the multiple complex micro-environments within the placenta and has shown promise for detecting a range of pregnancy complications at 3T [3,40]. We note that our definition of microenvironment includes both a singular tissue type, and combinations of multiple tissue types.

The structure of the placenta, and the complex interaction between structure and function involved in the aetiology of placental dysfunction, lends itself towards combined diffusion-relaxation MRI [31]. This is because the multitude of placental tissue types, e.g. villous trees and septa, and blood types, i.e. fetal and maternal, have a range of diffusion and relaxation MR properties that can be precisely quantified by measuring both simultaneously. For example [32], postulated that maternal blood has high T2 and low diffusivity, fetal blood has high T2 and high diffusivity, and tissue has low T2 and low diffusivity, and exploited this to separately quantify maternal and fetal circulations with T2-diffusion MRI and a three-compartment model. This showed sensitivity to early-onset fetal growth restriction [3]. Other work applied data-driven analysis to T2*-diffusion scans to reveal the T2*-ADC spectra, i.e. distribution of T2* and ADC values, showing promise for identifying dysfunctional placentas [39,40].

However, analysis and interpretation of diffusion-relaxation data is not straightforward. Such data is typically analysed with a model that makes fixed assumptions about tissue microstructure and can only calculate single averaged diffusivity and relaxation (e.g. T2, T2*) parameters per voxel [32]. Alternatively, model free approaches loosen this restriction to instead calculate distributions of the diffusivity and relaxation. However, these approaches are require many time-consuming MRI acquisitions and are highly sensitive to measurement noise [6].

An unsupervised learning approach, InSpect [39,41], offers advantages over standard diffusion-relaxation analysis approaches. It can parsimoniously identify distinct components in the data which potentially reflect distinct tissue microenvironments [39]. The MR properties and relative fractions of these components are potential biomarkers, which InSpect identifies without imposing an explicit tissue model. InSpect has shown promise for identifying dysfunctional placentas [39].

However, thus far InSpect has only been demonstrated on very limited data sets. Here we evaluate the potential of T2*-diffusion InSpect in a substantial data set of 78 healthy pregnancy image sets. We reveal and map detailed placental microenvironments, and the developmental pathways of these microenvironments during pregnancy.

## 2 Methods

We first describe our cohort recruitment and data acquisition protocol. We then specify our analysis approach in detail, starting with a brief background on continuum modelling in the placenta, then detailing how we applied an unsupervised learning method to identify and map placental microenvironments. We then explore and quantify how the relative fractions of placental microenvironments change over gestational age.

### 2.1 Participants

Participants were recruited as part of two ethically approved prospective cross-sectional studies at St Thomas’ hospital in London between 2016 and 2022 (Placenta Imaging Project [PIP], REC REC16/LO/1573; and Congenital Heart Disease Imaging Programme [CHIP], 21/WA/0075). Exclusion criteria common to both studies included maternal age below 16 or over 55 years of age, inability to give informed consent, BMI over 30 kg m^−2^, contraindications to MRI such as implants, pacemakers or claustrophobia, known fetal chromosomal anomalies and multiple pregnancies. Clinical details including medical history and outcome information including gestation at birth, birth weight centile, type of delivery, and neonatal outcomes were obtained. These were used to identify participants with uncomplicated pregnancies at the time of the scan - those with no evidence of pre-eclampsia, fetal growth restriction, fetal cardiac or neurological anomalies. Scans were subsequently excluded if such conditions or anomalies were newly diagnosed between the scan and delivery, if the pregnancy led to a delivery before 37 weeks gestational age, or if there were any notable incidental findings related to the fetus or placenta reported during imaging.

Our final cohort thus comprised 69 healthy pregnant participants, with 9 participants scanned at two timepoints during the course of their pregnancy, yielding 78 total scans. The mean GA at scan was 30.21 ± 4.63 [15.72, 38.29], maternal age 34.31 ± 4.09 [24.43, 45.13] years, maternal BMI 22.97 ± 3.02 [18.21, 32.32] kg m^−2^, 88 % of participants were white and 12 % non-white.

### 2.2 MRI scanning

We acquired placenta MRI data on our cohort with a previously reported T2*-diffusion sequence [40] that appends gradient echoes to a dMRI protocol specifically tailored for placental imaging [38]. We scanned the participants on a clinical Philips Achieva 3T scanner using a 32-channel cardiac coil consisting of 16 posterior and 16 anterior elements. Participants were scanned in supine position maintaining frequent verbal interaction as well as periodic life monitoring consisting of heart rate, oxygenation and blood pressure measurements. The data was acquired coronally to the mother using a multi-echo diffusion-weighted single-shot spin echo EPI sequence [19]. The 66 diffusion weightings varied from 0 to 1600 s mm^−2^ and the echo times (TEs) were 78, 114, 150, 186 ms, for a total of 264 contrast-encodings. The field of view was 300 by 320 by 84 mm, TR = 7 s, SENSE = 2.5, halfscan =0.6, resolution = 3 mm isotropic, and the total scan time was 8 min 30 s. We processed the data using in house tools including bias field correction, denoising, motion correction as previously described [13, 19]. We manually defined an ROI comprising the placenta and adjacent uterine wall (i.e. basal plate), in an identical procedure to our earlier works [39,40], on all scans. We chose not to separate the placenta from the basal plate in ROIs as delineating this boundary is difficult. Additionally, we wanted to include the basal plate in our analysis because it may contain valuable information.

### 2.3 MRI models

There are multiple approaches to analysing combined diffusion-relaxation data. The typical approach is microstructure modelling [1], where the number of tissue microenvironments is decided a-priori and fixed, and diffusion (e.g. diffusivities) and relaxation (e.g. T2*) parameters are estimated for each microenvironment. An attractive alternative is continuum modelling, which makes minimal assumptions about tissue structure, and calculates multidimensional correlation spectra - such as T2*-ADC spectra - rather than single values. These T2*-ADC spectra encode the distribution of T2* and diffusivity properties.

#### 2.3.1 T2*-ADC spectra

**Fig. 1:**
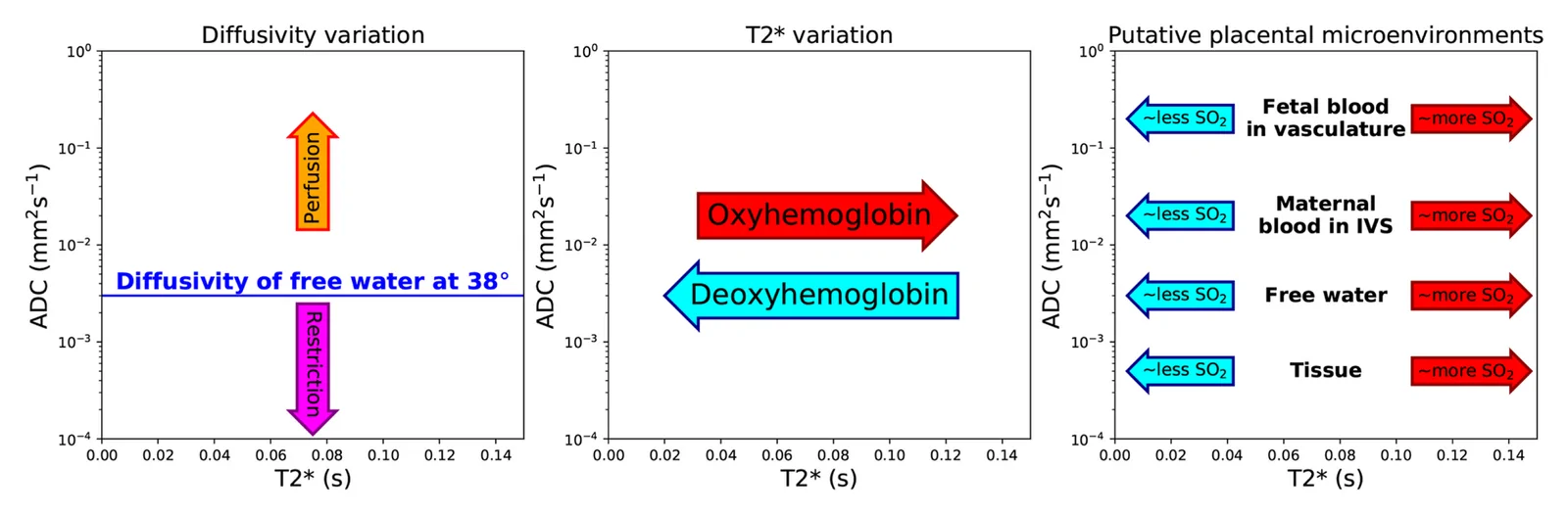
T2*-ADC spectra quantify diffusivity and T2*, which reflect microstucture and microcirculatory properties. A) The apparent diffusion coefficient (ADC) quantifies the average diffusivity rate of water in the sample, and can quantify perfusion and restricted diffusion. B) T2* is sensitive to oxyhemoglobin and deoxyhemoglobin levels, since paramagnetic deoxyhemoglobin leads to susceptibility changes in blood and hence dephasing of water in blood and tissue [34], and hence oxygen saturation levels. C) Applying the observations from A) and B) we can draw conclusions on the putative placental microenvironments that will be associated with different parts of the T2*-ADC spectra.

T2*-ADC spectra calculated from in-vivo MRI signals can be potentially associated with distinct tissue microenvironments. Fig. 1 uses the physical principles underlying diffusion and T2* contrast to hypothesise on the expected placental microenvironments associated with different areas of T2*-ADC spectra. Fig. 1A illustrates the ADC of free water at body temperature (3 × 10^−3^ mm^2^ s^−1^ [5]), and that ADCs higher than this imply water pseudo-diffusing due to an active process - i.e. perfusion, whereas ADCs lower than this imply diffusion restriction, e.g. by tissue structures. Fig. 1B illustrates the main effect underlying T2* contrast - the magnetic properties of oxyhemoglobin and oxyhemoglobin which reflect the level of saturated oxygen [34]. Whilst oxygenation is the main driver of T2* contrast, tissue composition, other magnetic field inhomogeneities, and the presence of other paramagnetic molecules are other factors [47]. Fig. 1C displays inferred positions of microenvironments within the T2*-ADC spectra. This includes two separates perfusion compartments - maternal and fetal. We make this distinction due to the structure of the placenta - maternal blood meanders in a tortuous path through the labyrinth-like intervillous space (IVS), whereas fetal blood perfuses in vessels. We note that fetal blood travels in different types of vasculature – aterial, venous, capillary – with different perfusion properties. We assume that these have the same perfusion properties, but acknowledge that this is a rough assumption.

### 2.4 MRI model fitting

We analysed our data with InSpect, an unsupervised learning technique based on continuum modelling that estimates a set of canonical T2*-ADC spectral components and corresponding mappings [39]. We hypothesise that each spectral component reflects a distinct tissue microenvironment. The corresponding mappings consist of voxelwise weightings that quantify the proportion of each voxel explained by each component, potentially reflecting the volume of each voxel occupied by individual tissue microenvironments. We fit InSpect to all scans simultaneously; the inferred T2*-ADC spectra were therefore shared across all participants. We specified 7 InSpect components when fitting for consistency with previous work, where this number was shown to best explain T2*-diffusion data with ROIs containing the placenta and basal plate [39]. InSpect code, including examples, is available at https://github.com/PaddySlator/inspect.

### 2.5 Analysing trends over gestation

We calculated the mean component weightings inside the placenta and uterine wall ROI for all scans. We plotted these mean weightings against gestational age and calculated the Pearson’s correlation coefficient for each component. We also fit a linear model with scipy for each component individually, with the gestational age the independent variable and the ROI-averaged component weighting the dependent variable, and hence calculated the slope (rate of change over gestation), intercept, R-squared, and p-value for the slope.

## 3 Results

### 3.1 InSpect output and MR properties of placental microenvironments

**Fig. 2:**
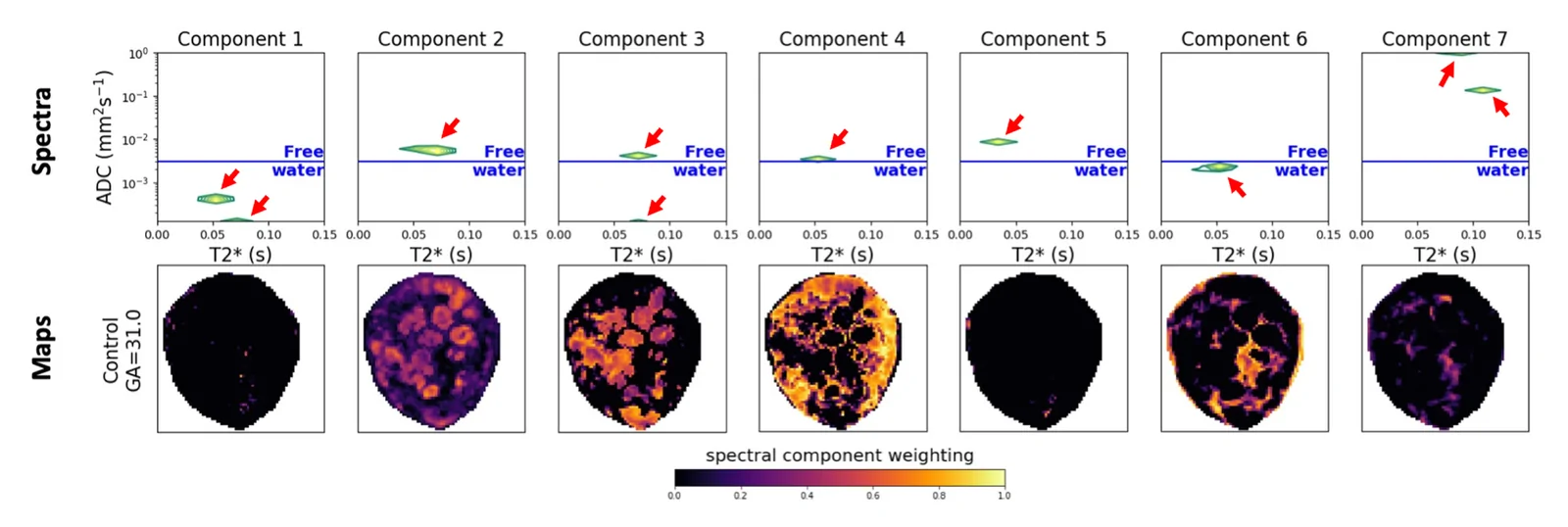
InSpect algorithm output for a single control participant, gestational age 31 weeks 0 days. Top row: canonical T2*-ADC spectra encoding the MR properties of each component. Bottom row: corresponding spatial maps showing the voxelwise weightings of each component. The maps encompass the placenta and uterine wall ROI, with everything outside the ROI white. Red arrows highlight the spectral peaks.

Fig. 2 illustrates the InSpect algorithm output for a single participant. The top row shows the canonical T2*-ADC spectra - which are shared across all scans since we fit InSpect to all scans at once - and the bottom row shows the corresponding mappings for a this single scan. The T2*-ADC spectra of the seven components (Fig. 2, top row) show that each component clearly has distinct MR properties. We explore these components in detail in the Discussion, but to briefly, and roughly, relate these components to the spectral areas identified in Fig. 1:

- component 1 may reflect very small tissue structures that markedly restrict water diffusion and have lower oxygenation
- component 2 may reflect maternal blood
- component 3 may reflect free water
- component 4 may reflect free water with lower oxygenation
- component 5 may reflect maternal blood with lower oxygenation
- component 6 may reflect tissue structures that somewhat restrict water diffusion and have lower oxygenation
- component 7 may reflect fetal blood with higher oxygenation

The corresponding spatial maps (Fig. 2, bottom row) reveal placental structures, such as the lobules (e.g. components 2 and 3) and surrounding septa (e.g. components 4 and 6).

### 3.2 Spatial maps of the placental microenvironments

**Fig. 3:**
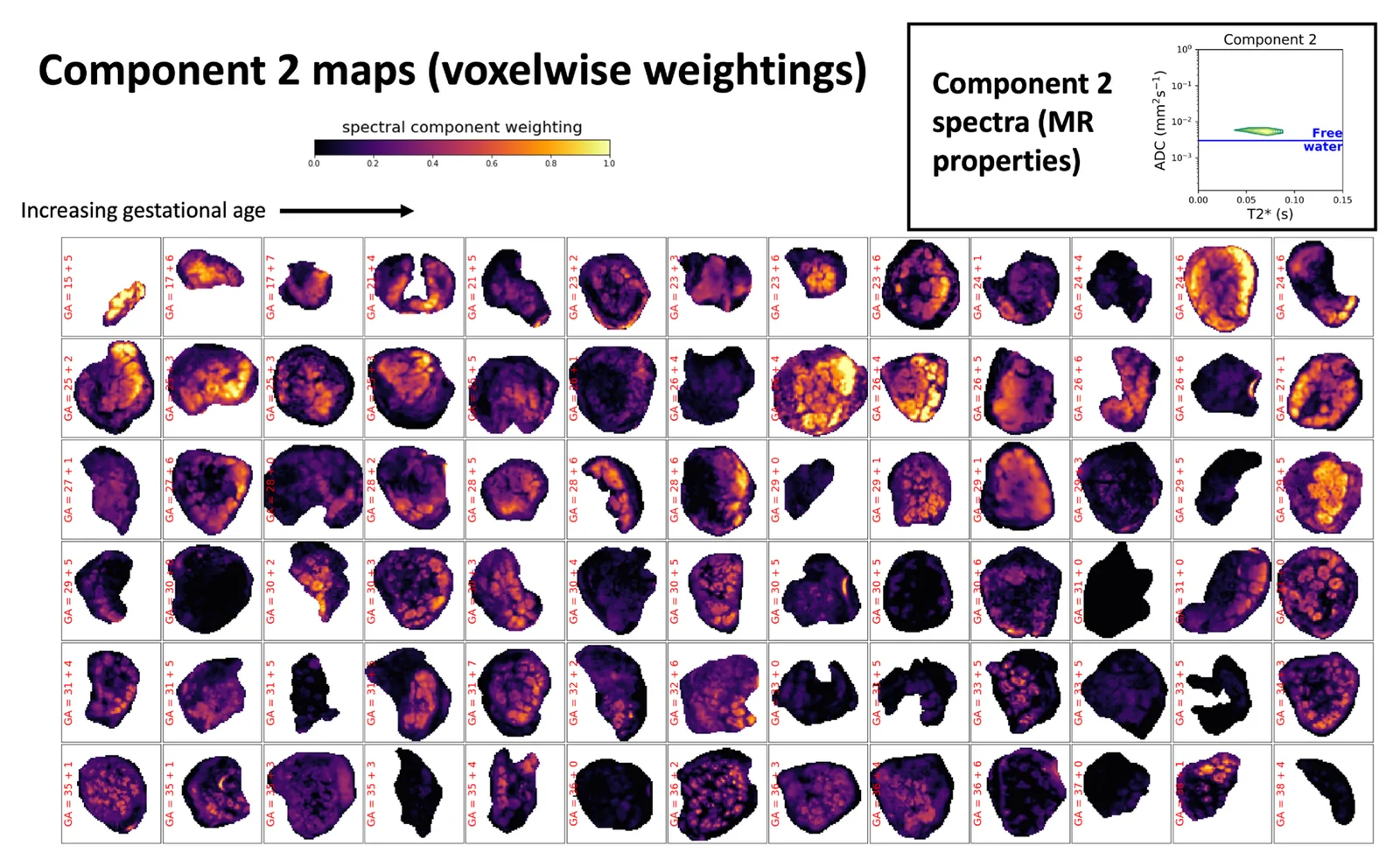
InSpect algorithm output for component 2. Top-right: canonical spectra encoding this component’s MR properties. Remaining images: corresponding maps, ordered by gestational age, quantifying the voxelwise weighting of this component for all scans. Each map shows a single representative slice; we chose the slice that most clearly shows a large region of the placenta. Note that the color scale is the same across all scans. See Fig. 4 and Supporting Figures S1-S5 for remaining component maps.

**Fig. 4:**
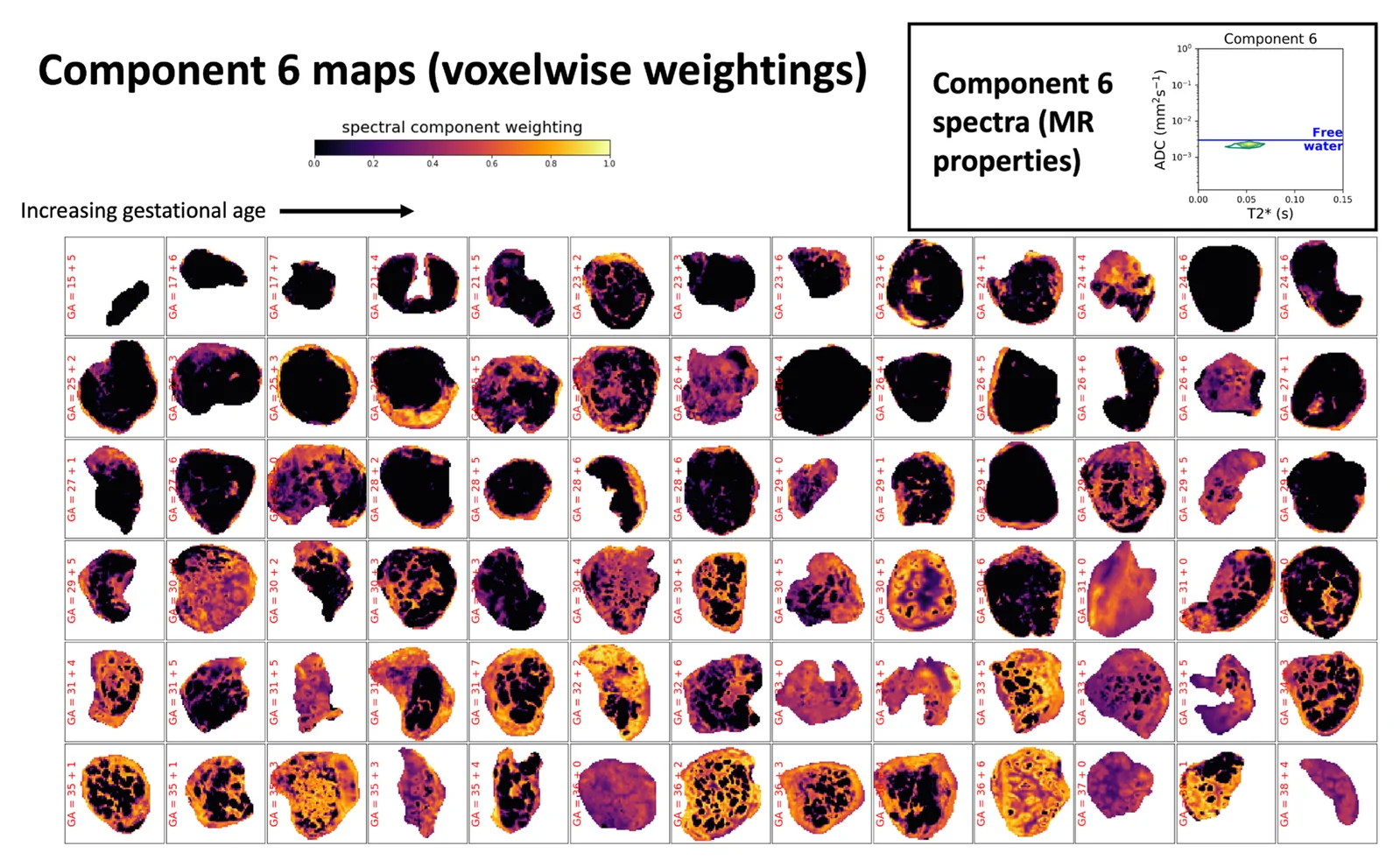
As Fig. 3 but for component 6.

**Fig. 5:**
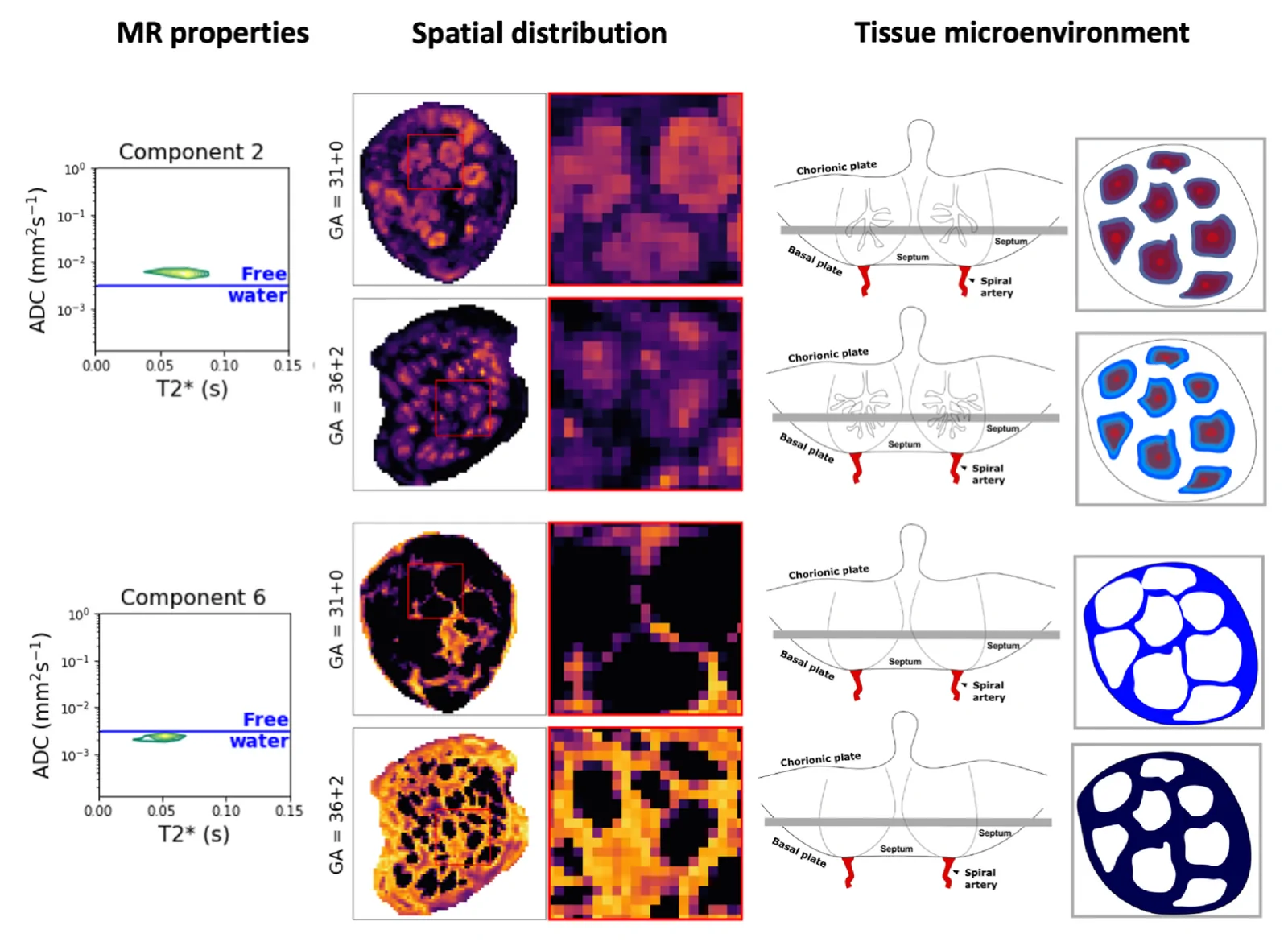
Our approach reveals small-scale placenta microenvironments. Left: component 2 and 6 spectra (“MR properties”) and corresponding example maps at two GAs (“spatial distribution”). Axial and coronal schematics (“tissue microenvironment”) highlight the placental regions with high weighting for each component.

Figs. 3 and 4 show maps for all 78 scans for two components which show apparent trends over gestation - components 2 and 6. We only show two components, each with opposite trends over gestation, in the main text for conciseness. See Supporting Figs. S1–S5 for the other components. The voxelwise weightings of component 2 decrease, on average, over gestational age, whereas the weightings of component 6 typically increase. This suggests that the proportion of the placenta comprised of these microenvironments decreases and increases over gestation respectively. In general, component 2 appears brightly in lobules at the start of pregnancy, then the weighting reduces. Component 6 appears mainly in the uterine wall in early pregnancy, then in later pregnancy forms an interstitial appearance, indicating that it resembles the empty spaces between lobules. Fig. 5 focuses on components 2 and 6, zooming in on substructures and linking them with placental microenvironments.

### 3.3 Changes in placental microenvironments over gestation

**Fig. 6:**
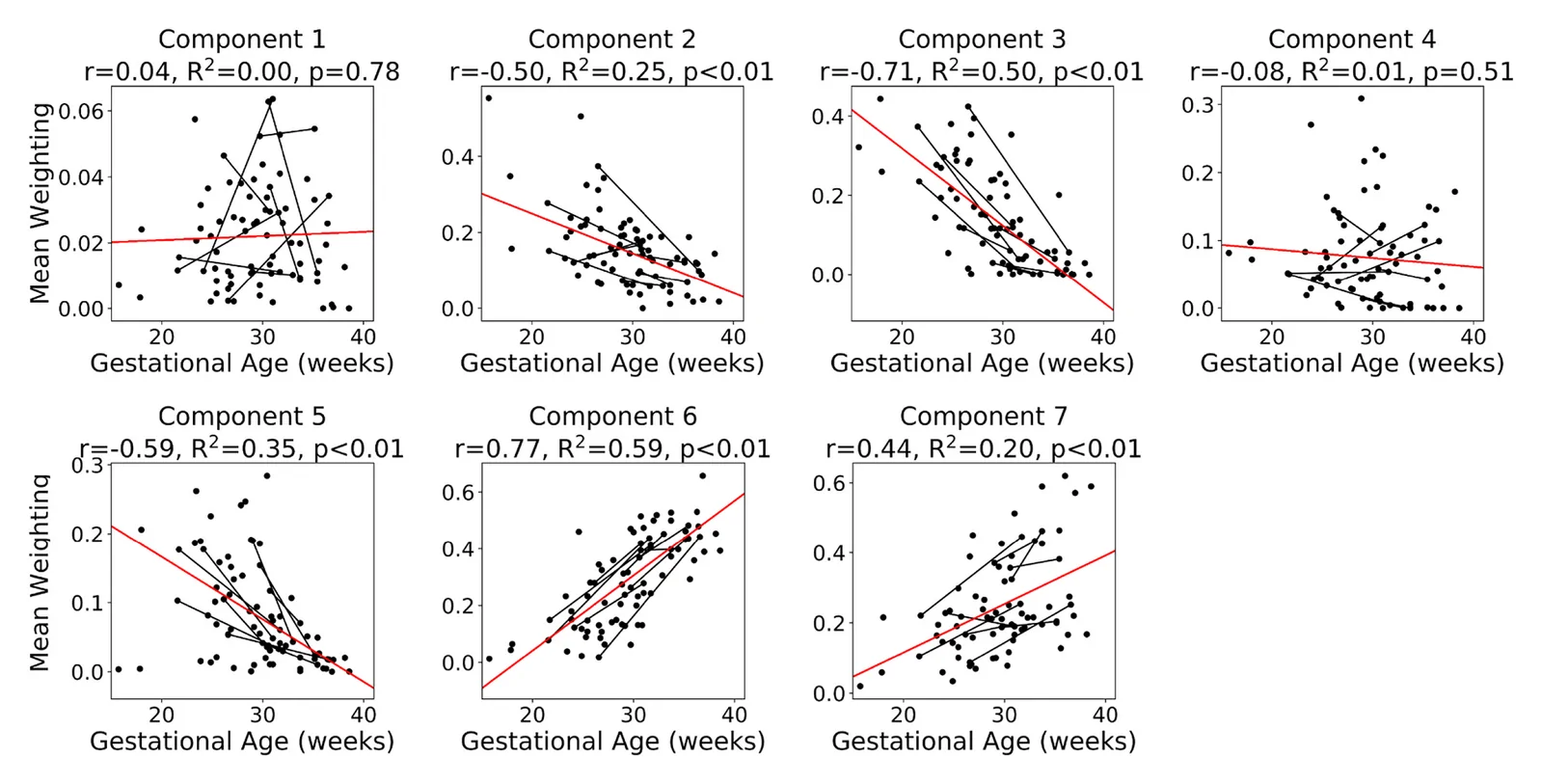
InSpect component weighting over gestation. Black lines joining data points depict participants who were scanned twice during their pregnancy. Red lines denote the linear fit. In the subplot titles r, R^2^, and p denote the correlation coefficient, R-squared of the linear fit, and p-value for the fitted slope respectively.

Fig. 6 plots the mean component weightings within the placenta and uterine wall ROI against gestational age and displays the fitted linear model. Table 1 displays the correlation coefficients, fitted coefficients, and associated statistics. All components except 1 and 4 show statistically significant (*p* < 0.01) changes in their ROI-averaged weighting over gestation (Table 1). Components 2, 3, and 5 clearly decrease across gestation, and components 6 and 7 clearly increase over gestation. The presence of these different trends over gestational age suggests that InSpect reveals components that reflect complementary placental maturation processes.

## 4 Discussion

We non-invasively probe placental structure and function throughout human pregnancy in unprecedented detail. Using combined diffusion-relaxation MRI and unsupervised learning, we identify and map seven putative placental microenvironments and track them longitudinally across gestation. Each microenvironment has a distinct T2*-ADC spectrum, which reflects its microstructural and microcirculatory properties, and spatial distribution, which reflects its arrangement throughout the placenta. The growth and decline in the relative proportions of each tissue microenvironment provides a window into placental development across gestation. Our work gives new insights into in-vivo placental structure and function, and how they progress during pregnancy. Our approach can potentially facilitate discovery of imaging biomarkers for identifying placental dysfunction and hence enable new techniques for prediction, diagnosis, and prognosis of pregnancy complications.

### 4.1 What tissue microenvironments are we measuring?

Each InSpect component comprises a canonical T2*-ADC spectrum, which tells us the MR properties of this component, and its corresponding map. We can hence interpret the specific tissue microenvironment that each component illustrates by studying its spectrum in relation to the physical principles underlying diffusion and T2* (e.g. Fig. 1), and maps. We emphasise components don’t have to neatly define placental compartments such as “fetal” and “maternal” or “intracellular” and “extracellular”; each microenvironment likely reflects a combination of distinct tissue compartments. We also note that some component’s spectral peaks appear to be overlapping, e.g. component 3 and component 4. However, these components still have marked differences in their voxelwise maps, suggesting that they still capture distinct tissue microenvironments. Our interpretation of each microenvironment, starting from the observations about each component we made in Fig. 1 and the Results section, is as follows.

1. Component 1 has two peaks with diffusivity much lower than free water, and T2* 0.05–0.07s. It has low overall weighting but is most prominent in the gaps between placental lobules. *We conclude that component 1 reflects a tissue microenvironment where diffusion is restricted, perhaps cellular structures typically located in the septa. The overall low weighting of this component may reflect the overall low levels of restricting tissue structures in the placenta*.
2. Component 2 has a single peak with ADC slightly above free water (~5 × 10^−3^ mm^2^ s^−1^) and reasonably high T2* (0.07 s). It is prominent in placental lobules, often more so in the lobule centres close to the spiral artery inlets. *Component 2 likely represents maternal blood slowly perfusing and percolating - faster than free water but slower than perfusion in vasculature - through tortuous intervillous space*.
3. Component 3 has two peaks, with one peak close to free water and one with very low diffusivity. Both peaks have T2* ~0.07 s. It is prominent in the space within the placental lobules typically attributed to the intervillous space. *Component 3 likely reflects water in tissue and/or maternal blood freely diffusing or very slowly perfusing within the intervillous space within the placental lobules*.
4. Component 4 has a single peak with diffusivity very close to free water (~3 ×10^−3^ mm^2^ s^−1^) and T2* ~0.05 s. It is prominent in the gaps between placental lobules. Component 4 likely reflects water in tissue and/or maternal blood freely diffusing in the septa. Its lower T2* compared to component 3 may reflect the lower oxygenation of maternal blood in these areas.
5. Component 5 has a single peak with ADC higher than free water (~1×10^−2^ mm^2^ s^−1^) and low T2* (0.03 s). It is prominent at the edges of placental lobules, often in a ring shape. *Component 5 likely reflects lower oxygenated maternal blood slowly perfusing and percolating through intervillous space*.
6. Component 6 has a single peak with ADC slightly below free water (~2.5 × 10^−3^ mm^2^ s^−1^) and T2* 0.05 s. It is prominent in the gaps between placental lobules. *Component 6 likely reflects water that is somewhat restricted in the septa*.
7. Component 7 has two peaks with high T2* (0.08 s) and diffusivity much higher than free water. It is prominent in the uterine wall and, later in gestation, throughout the placenta. *Component 7 likely reflects fast perfusing and highly oxygenated fetal and/or maternal blood*.

These microenvironments and their spatial distributions are consistent with expected placental structure and function. For example, microenvironments with diffusivity slightly higher than free water, such as component 2, are typically located in the centre of lobules where maternal blood perfuses and percolates through intervillous space (e.g. Fig. 5 top panel). On the other hand, microenvironments with lower diffusivity, such as component 6, are located in areas with more tissue structures such as the septa (e.g. Fig. 5 bottom panel).

Three components (2, 3, 5) show a clear decrease over gestation and two (6, 7) show an increase, suggesting that a range of complementary placental maturation processes are captured by our approach. These changes across gestation are consistent with trends observed in other modalities. For example, the decrease over gestational age in components 2 and 3 - which we hypothesise reflect different aspects of maternal blood - could reflect the rapid growth in the volume of terminal villi in the second half of pregnancy, as observed in stereology [22], with this causing a reduction in the volume of intervillous space within which maternal blood can perfuse and percolate. Component 5 - which likely corresponds to deoxygenated maternal blood - decreases over gestation. This may reveal the increasing oxygen demands of the fetal circulation during pregnancy. Component 6 - which likely corresponds to septa - increases over gestation. We speculate that this may reflect an increase in fibrous structures in the spaces between placental lobules.

### 4.2 Our method provides potential biomarkers

By quantifying the prevalence and spatial distribution of placental microenvironments our work gives detailed insights into normal placental development during pregnancy and identifies potential non-invasive biomarkers of complications. Although we don’t include complicated pregnancies in this study, by quantifying the normative pathway of placental maturation over gestation we provide a baseline that can potentially be used to compare against to identify dysfunctional placentas.

Our analysis enables more detailed insights than can be gained from parameters such as T2* and ADC. Whilst these both show sensitivity to pregnancy complications, they average over multiple structural and functional factors and don’t have an obvious biophysical or physiological interpretation. Our identified detailed and specific tissue microen-vironments relate more closely to specific tissue microenvironments and can hence enable more detailed insights into pregnancy complications and interpretable conclusions. This is supported by the observation that some components go up over gestation whereas some decrease - showing that we are sensitive to complementary maturation processes.

### 4.3 Limitations and future work

We emphasise that the connections we draw between InSpect components and distinct microenvironments are putative. An important area for future work is to independently validate these links. However, validation is complicated as there are multiple confounding factors, for example, T2* doesn’t simply measure oxygenation but is also affected by blood flow, blood volume, and the presence of paramagnetic molecules [27,47]. Moreover there are huge physiological changes in the placenta during delivery, such as the draining of the blood circulations and the degeneration of the maternal side of the placenta. Validation will therefore not be possible for all microenvironments - since we are measuring in-vivo physiological processes such as blood flow and oxygenation. However, we may be able to correlate some in-vivo measures with imaging of delivered placentas, e.g. with microCT [4,48], although another challenge that will need addressing is co-registration of the in-vivo and post-delivery data.

The InSpect component spectra we identify are somewhat different to those we identified in previous work [39]. This is expected to some extent, as InSpect is a data-driven method. Therefore, given new data, InSpect will infer new T2*-ADC spectra, which complicates comparisons between tissue microenvironments and the interpretation and useability of the method. An option is to fix the T2*-ADC spectra, e.g. to the spectra we inferred from a large cohort of uncomplicated pregnancies in this paper, and only calculate the corresponding maps in subsequent placental scans. These maps could then be used to assess the deviation from normality for the scanned placenta. This has the additional advantage that calculating maps given fixed spectra is much quicker than running the full InSpect algorithm, potentially enabling results to be available at scan time.

In this study, one person manually drew the ROIs. In future, we will explore intra- and inter-rater variability and explore how robust the InSpect maps, spectra, and trends over gestation are to the choice of ROI.

The key area for future work is to move towards testing, reproducing, validating, and deploying the potential imaging biomarkers produced by our approach in additional cohorts. We utilised 78 scans in this study, with each scan containing tens of thousands of voxels. Whether this is enough to reliably calculate the spectra and maps, and, crucially, to uncover differences between cases and controls, remains an open question. The clear first step towards this is to extend our approach to include participants with pregnancy complications and compare our InSpect metrics with other imaging biomarkers, such MRI-derived T2* and ADC, ultrasound measures and other clinical measures such as blood pressure.

Comparisons between control and patient cohorts could be made through case-control studies or normative modelling, an alternative paradigm where statistical inferences are made by comparing measurements from individual subjects with a reference model [35], an example reference model being the fetal growth charts for estimated fetal weight from ultrasound measurements [24]. For case control studies, we would need an expanded study including participants diagnosed with a pregnancy complication. Normative modelling would require orders of magnitude more participants - both controls and complicted pregnancies - with increased geographical and ethnic diversity. The ultimate goal is to establish quantitative baselines against which placentas can be compared against, to support clinical decision making and hence improve pregnancy outcomes.

To conclude, we non-invasively reveal small-scale placenta structure and function during human pregnancy by identifying and mapping multiple placental microenvironments and tracking their progression throughout gestation. Each microenvironment has different micro-structural and microcirculatory properties even though we don’t impose an explicit microstructural model. Tissue microenvironments match with known placental structure, revealing and tracking detailed and specific microstructural and microcirculatory features over pregnancy. By demonstrating direct observation of micro-scale placental structure and function, and revealing clear trends across pregnancy, our work contributes towards the development of robust imaging biomarkers for pregnancy complications, and the ultimate goal of a normative model of placental development that enables clinicians to make improved treatment and management decisions.

## Supplementary Material

**Appendix A. Supplementary data**

Supplementary data to this article can be found online at https://doi.org/10.1016/j.placenta.2023.11.002.

Supporting Figures and Tables

## Figures and Tables

**Table 1:** Statistical overview of ROI-averaged component weighting changes over gestation. Correlation coefficient (Pearson’s) is between each mean component weighting and gestational age. Intercept, slope, R-squared, and p-value are from fitting a linear model to the data, with the gestational age the independent variable and the ROI-averaged component weighting the dependent variable. The p-value is associated with the slope coefficient, and thus indicates whether the relationship between the gestational age and ROI-averaged component weighting is statistically significant.

Component	Correlation coefficient	Intercept	Slope (rate of change per week)	R-squared	p-value
1	0.04	0.02	1.26e-04	1.25e-03	7.81e-01
2	– 0.50	0.46	– 1.05e-02	2.49e-01	2.69e-05
3	– 0.71	0.71	– 1.95e-02	5.03e-01	5.39e-11
4	– 0.08	0.11	– 1.29e-03	7.17e-03	5.06e-01
5	– 0.59	0.35	– 9.05e-03	3.48e-01	2.85e-07
6	0.77	– 0.49	2.64e-02	5.88e-01	1.54e-13
7	0.44	– 0.16	1.38e-02	1.96e-01	2.48e-04
