# Supplementary material for: Non-invasive mapping of human placenta microenvironments throughout pregnancy with diffusion-relaxation MRI
Source: Placenta. Author manuscript; Available in PMC 2026 Oct 15. (PMC7619552; doi:10.1016/j.placenta.2023.11.002)
Supplement: Supplementary file 1 [file EMS219728-supplement-Supporting_Figures_and_Tables.pdf]

6. Supporting Figures and Tables

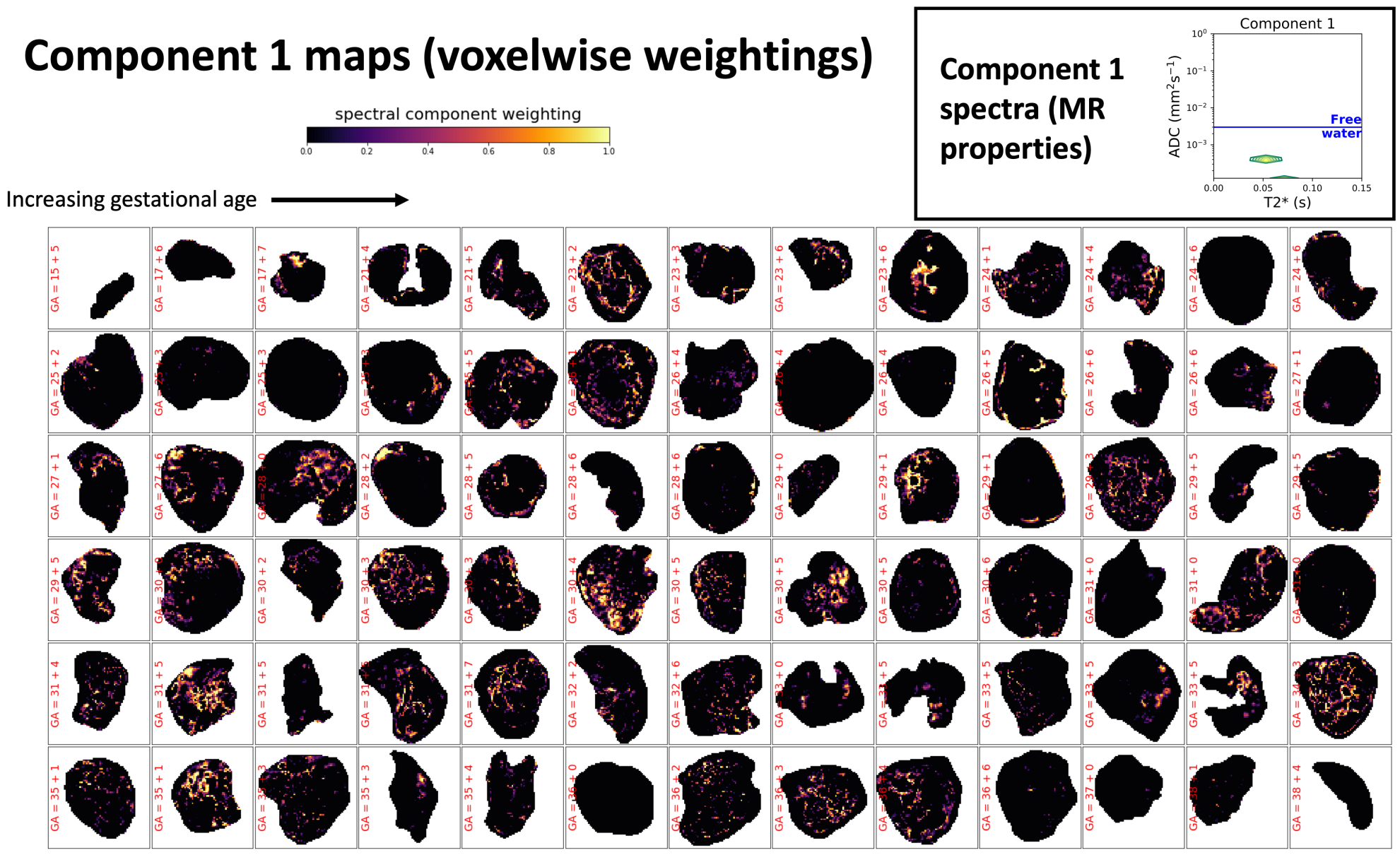

Figure S1: As Figure 3 but for component 1.

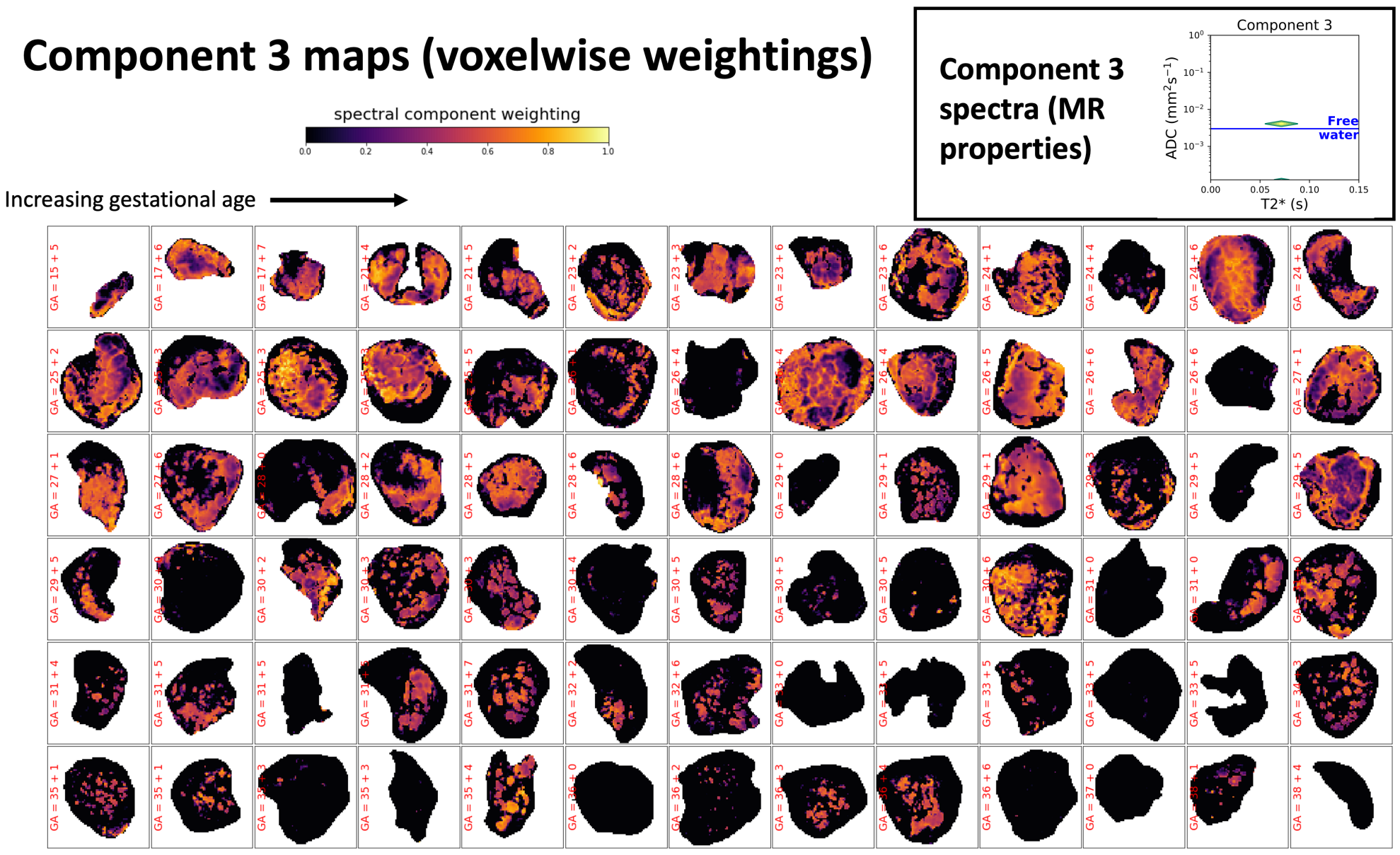

Figure S2: As Figure 3 but for component 3.

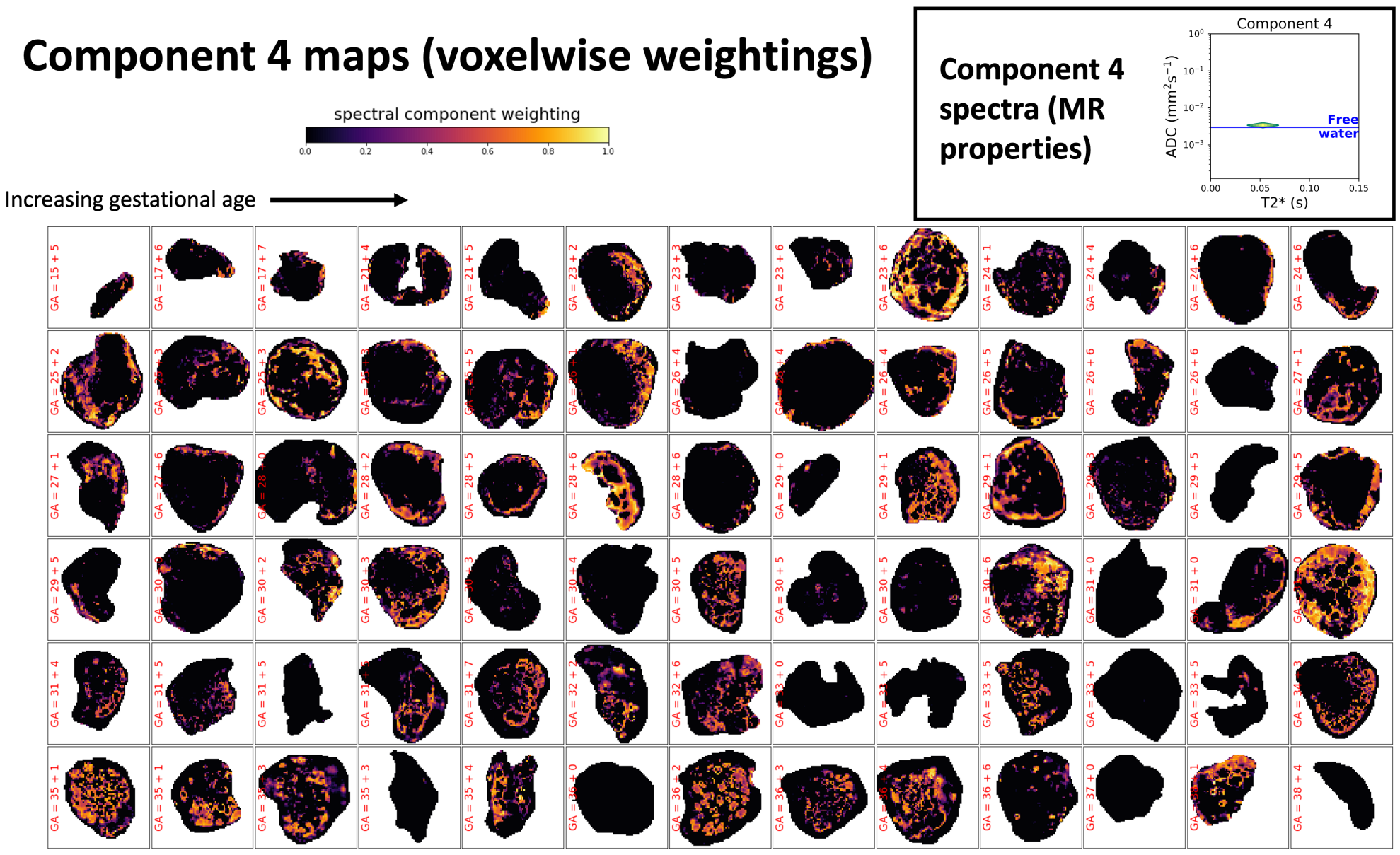

Figure S3: As Figure 3 but for component 4.

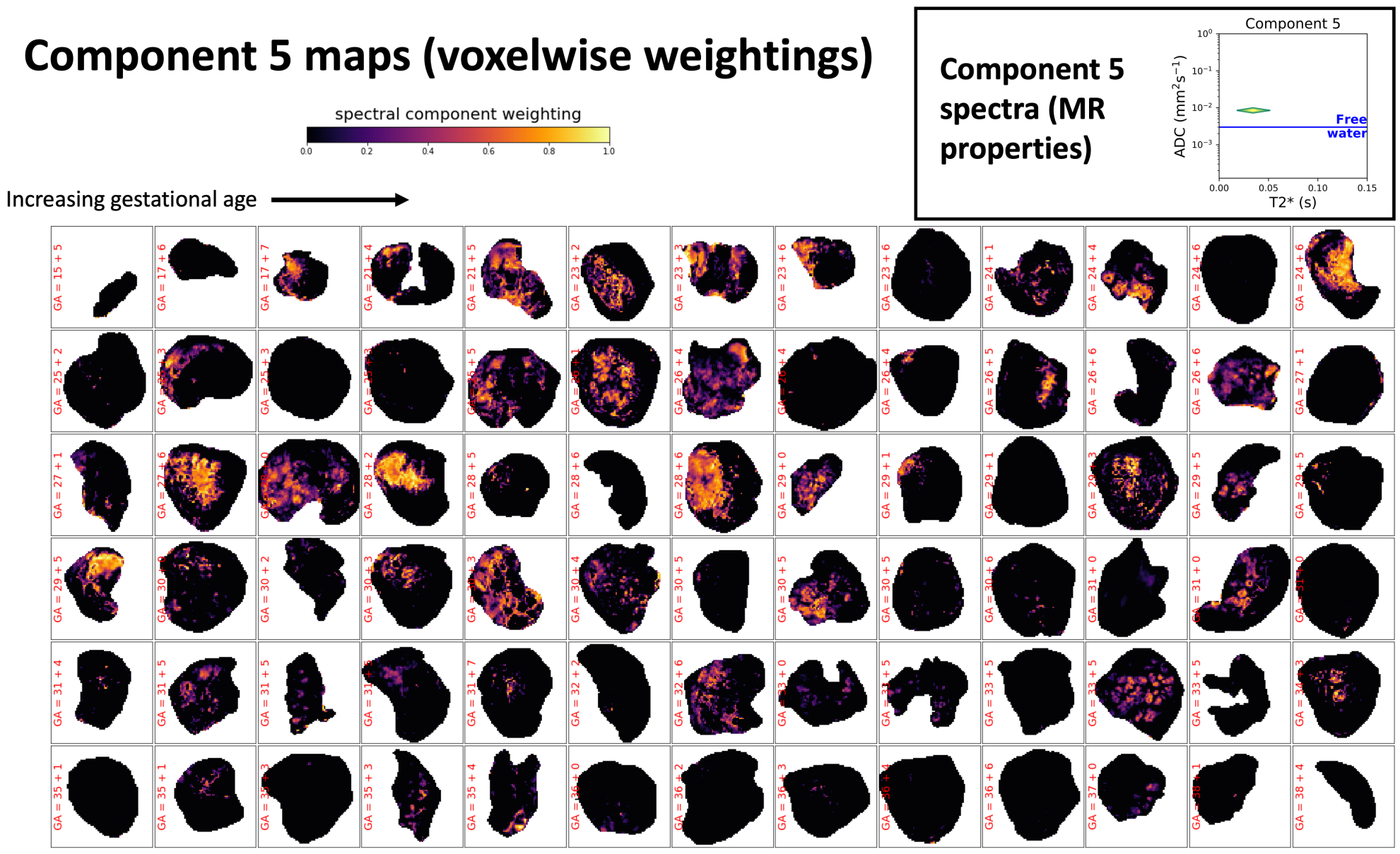

Figure S4: As Figure 3 but for component 5.

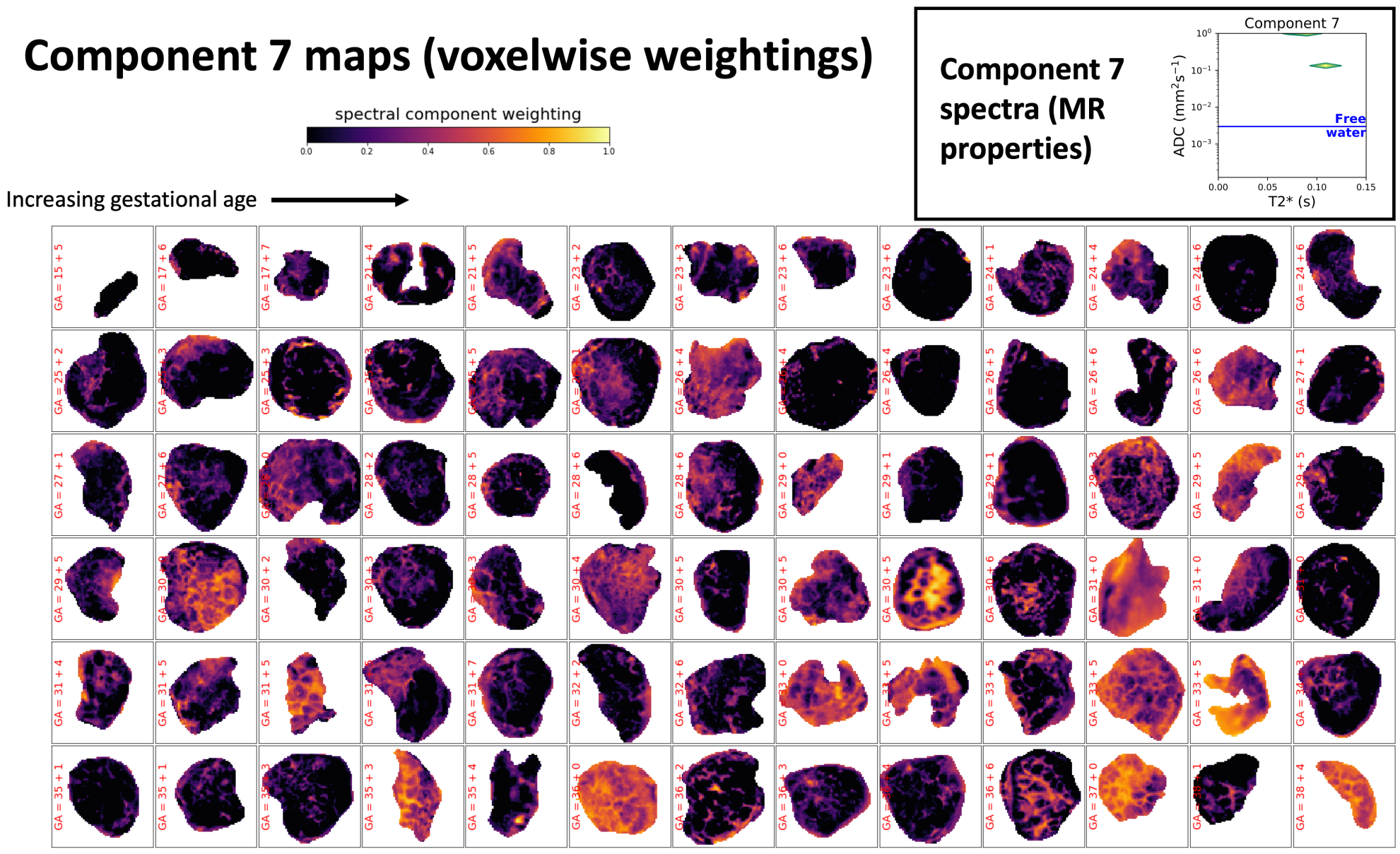

Figure S5: As Figure 3 but for component 7.
